# Doping colloidal bcc crystals — interstitial solids and meta-stable clusters

**DOI:** 10.1038/s41598-017-12730-8

**Published:** 2017-10-03

**Authors:** Ruben Higler, Joris Sprakel

**Affiliations:** Physical Chemistry and Soft Matter, Wageningen University, Wageningen, 6708 WE The Netherlands

## Abstract

The addition of a small amount of dopant impurities to crystals is a common method to tune the properties of materials. Usually the doping grade is restricted by the low solubility of the dopants; increasing the doping concentration beyond this solubility limit leads to supersaturated solutions in which dopant clusters dominate the material properties, often leading to deterioration of strength and performance. Descriptions of doped solids often assume that thermal excitations of the on average perfect matrix are small. However, especially for bcc crystals close to their melting point it has recently become clear that the effects of thermal disorder are strong. Here we study the doping of weak bcc crystals of charged colloids via Brownian dynamics simulations. We find a complex phase diagram upon varying the dopant concentration. At low dopant concentrations we find an interstitial solid solution. As we increase the amount of dopants a complex meta-stable liquid-in-solid cluster phase emerges. Ultimately this phase becomes meta-stable with respect to macroscopic crystal-crystal coexistence. These results illustrate the complex behaviour that emerges when thermal excitations of the matrix drive impure crystals to a weak state.

## Introduction

A common approach to tune the properties of crystalline solids is the process of doping; the introduction of interstitial atoms in a crystalline matrix with the purpose of changing the materials properties. The canonical example of this process is the addition of small amounts of carbon as an interstitial dopant in an iron matrix to toughen the material and create steel. The movement of Li-ions through an inorganic matrix in superionic solid-state batteries, such as Li_10_GeP_2_S_12_, is also a form of interstitial doping since the Li-ions move through the lattice via a percolating network of interstitial sites^1,2^. In the case of Fe, the addition of too much carbon, e.g. in cast iron, makes a very brittle substance. Clearly the properties of doped crystalline materials are highly dependent on the degree and distribution of dopants. The solubility limit of dopants is often very low; e.g. 0.022 wt% for carbon in *α*-Fe at 723 °C^3^. With the addition of additional dopants beyond this point precipitation of the alloy occurs^4^ and material properties degrade. Due to the small length scales inherent in the study of atomic materials, the process of crossing the solution limit; going from a low concentration of dopant to a supersaturated solid solution, has not been investigated on a single particle level. Models and experiments have found evidence for dopant clustering and the formation of dopant rich domains in supersaturated interstitial alloys^5–8^. However, due to the inherently short time and length scales involved in atomic materials, investigation into supersaturated solid solutions on a single particle level is very difficult. To overcome this limitation we explore the use of colloids as a model system for impure crystals; these systems are easily observed with microscopy techniques and show behaviour that in some cases is analogous to their atomic counterparts^9^.

While pure colloidal crystals have been studied in detail, much less is known about impure crystals. Binary systems, that is systems which consist of two particle species with a high size asymmetry such as doped crystals, show a complicated phase diagram identified using both experiments^10–12^ and simulations^13,14^. However, so far research has focused on hard-sphere colloidal systems, which only exhibit an face-centred cubic (fcc) structure. This is in contrast with many metals which have body-centred cubic (bcc) crystal symmetry, especially at conditions used for processing, temperatures at or slightly below their melting points^15^.

Colloidal systems of charged spheres where interactions take the form of long ranged repulsive Yukawa pair-potentials, show a much richer phase behaviour; exhibiting a low density bcc phase. This model for weak bcc crystals is akin to those found in electron systems^16^, neutron stars^17^, dusty plasmas^18^. These very soft bcc lattices are unique as their transition into the liquid is weakly first order^15^. Recent experimental work has evidenced the weak first-order transition and entropic stabilization of weak bcc crystals and shown that this leads to a failure of classical lattice theory^19^. Rather, strong thermal disorder and large correlated fluctuations govern the crystal physics. This has some unusual effects on the properties of the crystalline solid that, while ordered on average, displays some features typically associated with disordered solids, such as a elasticity that is governed by non-affine fluctuations, leading to a breakdown of the Born-Huang lattice dynamics^19–22^.

Since the elasticity in these thermally-disordered crystals becomes governed by non-affinity and the classical rules of crystal lattice dynamics fail, one may also expect that doped crystals would also behave anomalously. It is the weakness of these bcc crystals which also results in a unique behaviour when they are doped. We have previously shown that the large off-lattice fluctuations are the reason existing theoretical predictions for dopant dynamics break down^23^. Firstly the large thermal excitations of the bcc lattice, destroy the percolating path of interstitial sites and significantly restrict the motion of interstitial dopants through the crystal. Secondly, the lattice deformation that results from the introduction of a dopant mediates an effective attractive interaction between the dopants. At the low doping levels studied, small clusters were observed as a result of this; again restricting the diffusion of dopants, this time due to the formation of clusters. So far only low doping levels have been investigated; this raises the inevitable question of what happens when doping concentrations reach supersaturated levels, where for atomic solids it is known that material properties rapidly change — such as a notably lower rate of diffusion similar to what we observed in colloidal systems^24^.

In this paper we study, using Brownian dynamics simulations, the behaviour of weak bcc crystals of repulsive Yukawa particles, doped with a wide range of doping fractions. We identify a transition, with increasing number of added dopants, from a interstitial solid-solution phase, ***ISS***, to a meta-stable phase of phase-separated liquid pockets, ***XLP***. This phase is a coexistence of a crystalline base particles phase with spherical pockets of liquid dopants. At higher dopant concentrations we find a second transition where the liquid-pockets fuse and a macroscopic crystal-liquid, ***XL***, coexistence emerges. As we reach the highest dopant concentrations, the local volume fraction of the dopant phase crosses its melting point and we find a crystal plus meta-stable liquid, ***XML*** phase. The meta-stable liquid will, given enough time, crystallise resulting in complete crystal-crystal phase separation, ***XX***. These results give new insights into the complex phase behaviour of impure colloidal crystals.

## Results and Discussion

**Figure 1:**
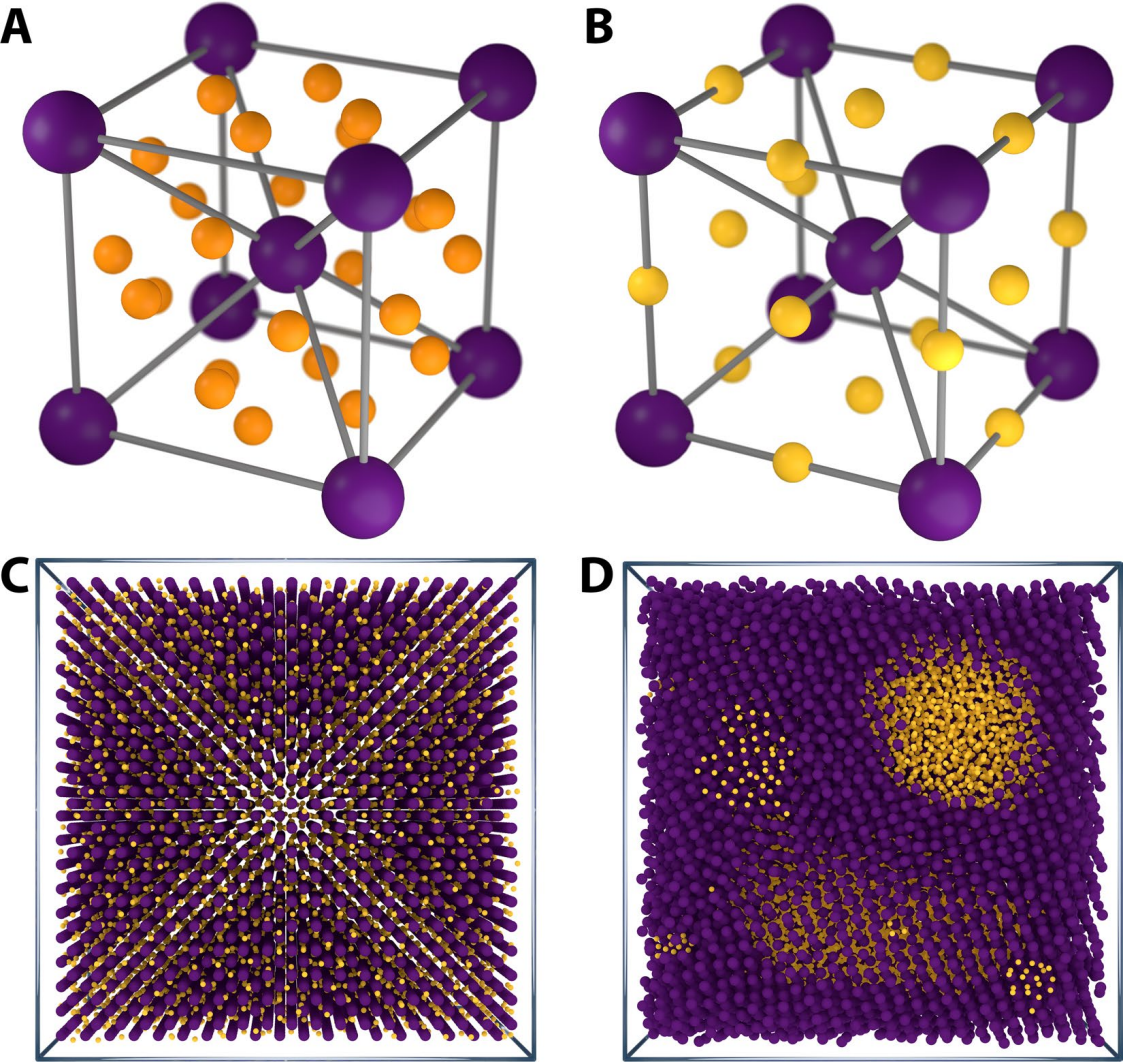
bcc unit cell & influence of base crystal. (**A**) Computer rendering of a body-centered cubic (bcc) unit cell (purple, large spheres) and locations of the tetrahedral interstitial sites (orange, smaller spheres); of which there are four on each face of the unit cell (twelve per unit cell). (**B**) Computer rendering of a body-centered cubic (bcc) unit cell (purple, large spheres) and locations of the octahedral interstitial sites (yellow, smaller spheres); of which there is one on each face of the unit cell and one on each edge (six per unit cell). (**C**) If we ignore the dynamics of the base crystal particles the dopant particles are distributed homogeneously throughout the crystal. (**D**) Due to matrix mediated effective attractive interactions between dopants we observe clusters and phase separated liquid pockets in systems where we do take into account the influence of base particle dynamics.

We simulate a base crystal consisting of 13718 particles with diameter, *σ*_*base*_ = 1.8 *μm*, at a volume fraction *ϕ*_*base*_ = 0.10 inside a box of length *L*_*x*_ = *L*_*y*_ = *L*_*z*_ = 41.57 *σ*_*base*_ with periodic boundary conditions. These bcc crystals are doped with particles with diameter *σ*_*dopant*_ = 0.9 *μm*. The resulting size ratio $\rho =\frac{{\sigma }_{dopant}}{{\sigma }_{base}}=0.5$ is close to that of carbon in iron^25^. The concentration of dopants is our variable, expressed as a fraction of the total available tetrahedral interstitial sites (Fig. 1) in the base crystal which are occupied; the interstitial fraction, ***IF***. Since there are 12 tetrahedral interstitial sites per unit cell in a bcc crystal ***IF*** = 1.0 would equal 82308 dopants divided over 6859 unit cells.

In doped crystals the impurities will, if small enough, position themselves in the lowest-energy interstitial sites of the surrounding crystal matrix. In the case of a perfect bcc crystal we can identify two types of interstitial sites; tetrahedral (Fig. 1A) and octahedral sites (Fig. 1B), so named after the number of faces of the polyhedron created by treating the nearest-neighbour matrix particles as vertices. These sites form the local minima in the potential field created by the base crystal particles. Dopants prefer to reside in the tetrahedral sites because these have the lowest potential energy of the two different types. In bcc crystals dopant diffusion between tetrahedral interstitial sites has classically been described as a thermally-activated hopping process governed by the energy barrier, *U*_*A*_ between two adjacent sites. Within this approach it is assumed that the potential energy field that a dopant experiences is static and prescribed by a perfect lattice, and that interactions between dopant particles are negligible. In a previous paper, we showed that dopant dynamics in soft crystals approaching their melting point, *ϕ* ~ *ϕ*_*melt*_, can not be adequately described by theories based on the assumptions. Long-time diffusion coefficients for the dopants are two orders-of-magnitude lower than those predicted based on the idealised energy landscape. However, in both situations diffusion through the lattice is still faster than hard sphere systems^10^. When we take thermal motion of the base crystal into account, the well-defined local minima in the potential energy landscape disappear due to strong excitations of the marginally-rigid crystal, such that well-defined interstitial sites can no longer be identified from snap-shots of the structure. The percolating path of tetrahedral sites is broken, which prevents interstitial particles to diffuse effectively throughout a bcc crystal; significantly lowering their long-time diffusion coefficients.

Another factor slowing down the dopant diffusion is the emergence of attractive dopant-dopant interactions; even though all pair interactions in our system are repulsive. The introduction of dopants into the crystal leads to a small deformation of the lattice. The lattice strain is minimised by clustering dopants^26^, resulting in emergent elastic interactions between dopants. These effects have recently been proven in experiments on colloidal hard sphere crystals; showing how a stress field emerging from a point defect causes relatively long ranged defect-defect interactions^27^.

**Figure 2:**
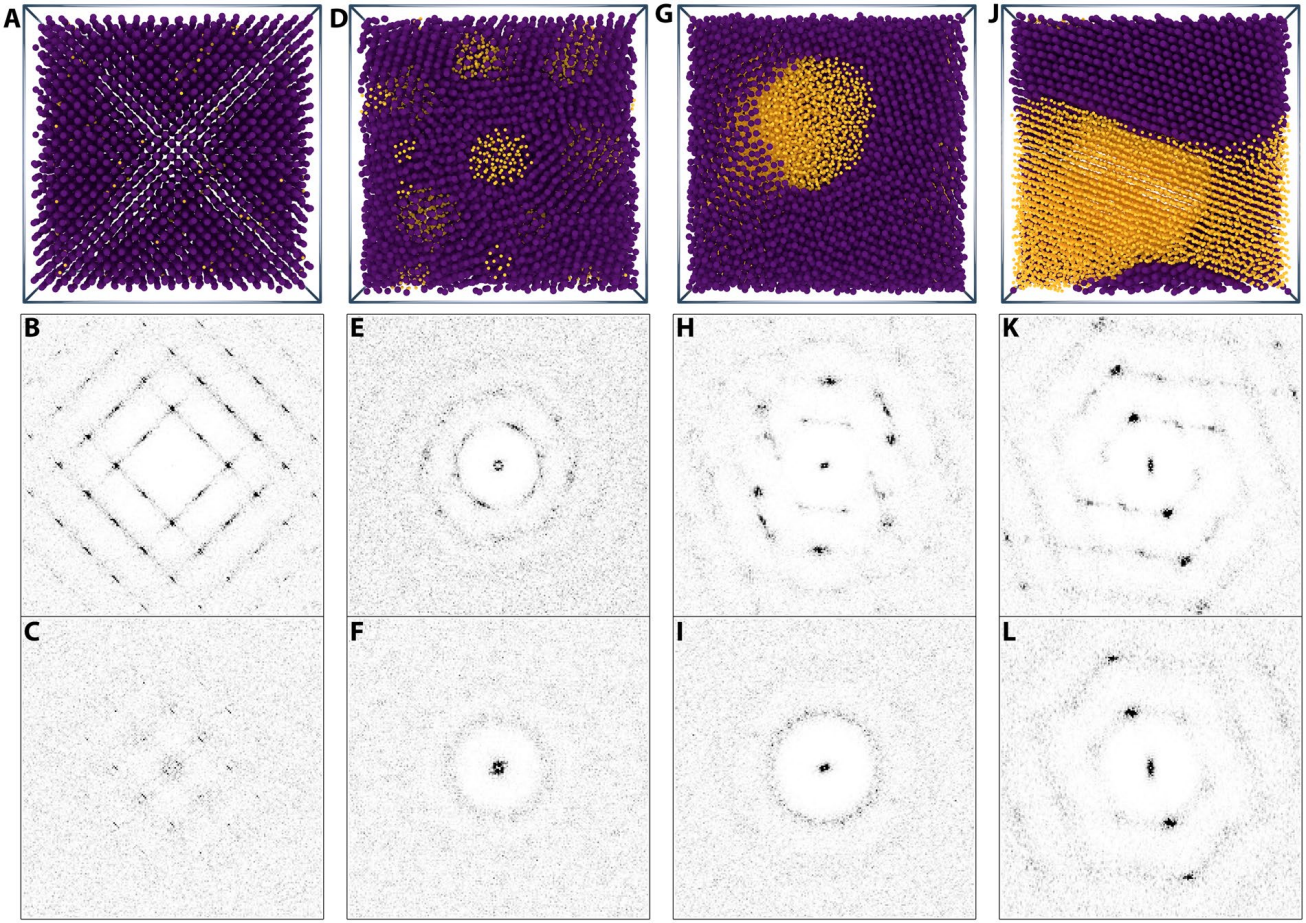
Increasing dopant concentration leads to phase separation. (**top row**) Computer renderings of final simulation snapshots of simulated doped bcc crystals. With base crystal particles in purple and dopants in yellow. From left to right the dopant interstitial fraction, ***IF***, increases (***IF*** = 0.01, 0.05, 0.20, & 0.25) and transitions from an interstitial solid solution, to phase separated liquid pockets of dopants, and finally towards a fully phase separated crystal-crystal system. (**middle row**) Two-dimensional structure factors *S*(*q*_*x*_, *q*_*y*_) calculated from particle centre-of-masses for base particles only. Even though the base crystal lattice is highly disrupted by the liquid clusters, the visibility of Bragg peaks over the entire ***IF*** range indicates the continual presence of local crystalline domains in the system. (**bottom row**) Two-dimensional structure factors *S*(*q*_*x*_, *q*_*y*_) calculated from particle centre-of-masses for dopant particles only. There is clear transition from visibly though smeared-out peaks in the interstitial solution, ***ISS***, phase at low ***IF*** into a dopant liquid pocket, ***XLP***, phase which finally at the point of full phase separation crystallises.

**Figure 3:**
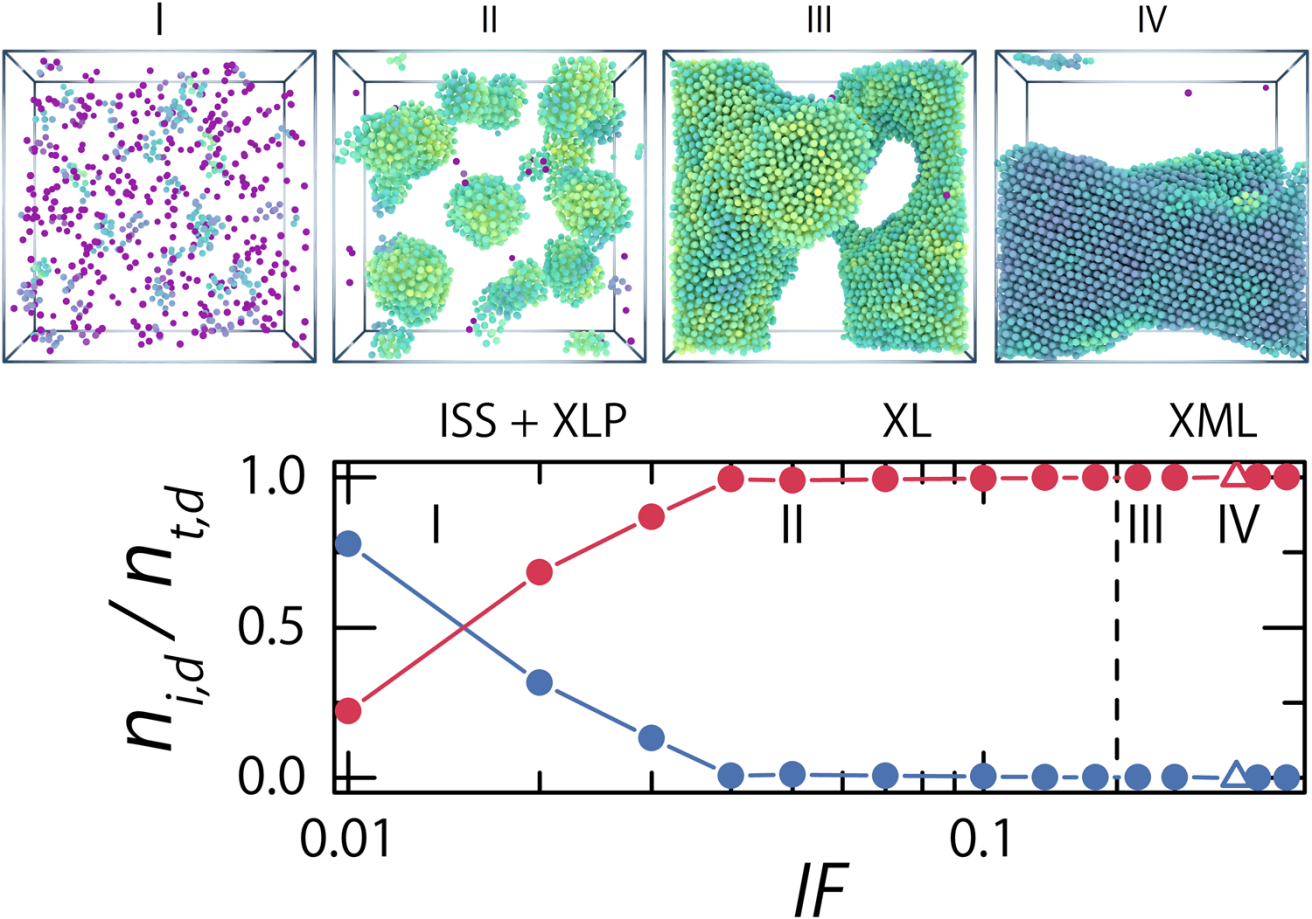
Phase behaviour of strongly doped crystals. (**bottom row**) We show the evolution of the fraction of interstitial dopants (blue circles) and phase separated dopant particles (red circles) as a function of the total amount of dopant particles in the system; expressed as the fraction of tetrahedral interstitial sites filled at the start of the simulations, ***IF*** = 0.01, 0.02, 0.03, 0.04, 0.05, 0.07, 0.10, 0.125, 0.15, 0.175, 0.20, 0.25, 0.27, & 0.30. The triangular symbol indicates the one sample we found which showed predominately bcc symmetry in the dopant phase. We describe four distinct regimes as indicated with roman numerals in the figure. (**I**) A transitional regime from a phase dominated by dopants acting as an interstitial solid solution towards a phase of dopants phase separated into liquid clusters or into a single liquid phase (**II**). (**III**) In this regime the volume fraction of the dopant phase has increased above melting point (*ϕ*_*m*_ ≈ 0.031) for a dopant only system (dashed vertical line) and we find meta-stable liquid, crystalline (**IV**) or mixed dopant phases. (**top row**) Visual illustration of the different regimes identified. We have rendered the dopant particles at twice their size and coloured them according to their bond-orientational order parameter, ${\bar{q}}_{6}$.

In our simulations, these effects are evident when we compare dopants in a thermal and a static base crystal. With a static base crystal we find the dopants distributed homogeneously throughout the bcc lattice over all available interstitial sites. Switching the thermal excitations of the base crystal on results in clustering of dopants due to the emergence of attractive forces between the dopants (Fig. 1C and D). So far we have only investigated systems in the low doping limit, thereby minimising dopant-dopant interactions. As the amount of dopants increases a complex phase behaviour appears. The doping degree is expressed as the fraction of tetrahedral interstitial sites that have a dopant particle present at the beginning of our simulations, the interstitial fraction ***IF***, ranging from 0.0 for zero doping to 1.0 when all tetrahedral interstitial sites (12 per unit cell) are filled. At the lowest ***IF*** we simulate, 0.01, the dopants are spread evenly over the entire bcc crystal; an interstitial solid solution, ***ISS***; similar to what has been found for doped hard sphere crystals^10,28,29^. In doped hard sphere systems when the dopant diameter, *σ*_*d*_, is smaller or equal to the largest interstitial site — octahedral sites for fcc crystals, *σ*_*IS*_ — i.e. *σ*_*d*_ ≤ *σ*_*IS*_, there is no lattice strain due to the introduction of the interstitial impurity. In this case, in absence of enthalpic interactions, there is no cause for clustering. If the hard-sphere interstitial is larger than the interstitial site, lattice strain accompanies the doping, for example for the particular case of self-interstitials, clustering occurs at sufficient doping fractions^30^. By contrast, for the Yukawa system, even dopants that geometrically fit into the interstitial void spaces exert enthalpic forces onto the surrounding matrix, causing a deformation of the crystal and providing a mechanism for attractive dopant-dopant interactions. Indeed, we observe the presence of a few, small clusters of dopants even at the lowest ***IF*** (Figs 2A and 3 example I).

Based on an analysis of the averaged local bond-orientational order parameter ${\bar{q}}_{6}$ of the dopant-dopant structure, we conclude that the majority of these clusters have an internal bcc symmetry, as evident from the orientational bond-order parameter ${\bar{q}}_{6}\approx 0.4$ (see Methods), and therefor represent clusters of interstitial particles. A minority of clusters have ${\bar{q}}_{6}$ values in the range we expect for liquids (${\bar{q}}_{6}\approx 0.2$)^31^; these clusters consist of phase separated dopant particles forming a small liquid pocket in the bcc base crystal. The presence of dopants in two states is confirmed by the structure factor *S*(*q*_*x*_, *q*_*y*_) of both the base particles and dopants. The bcc symmetry of the base crystal is clearly reflected in the calculated diffraction pattern (Fig. 2B). For the dopants we see blurred bcc reflections, with a sharp peak in the middle; the result of dopant particles at their tetrahedral interstitial sites combined with the few small liquid-pockets (Fig. 2C). We note that the use of the orientational bond-order parameter only probes the local and static structure of the solid and has no bearing on possible anomalies that may emerge in these solids due to non-affinity in their mechanics and dynamics^19,32^. However, since these latter are not part of the topic of this study, the static bond order parameters provide us insight into the local particle symmetry.

With increasing ***IF*** the system transitions into a cluster phase characterised by pockets of dopant liquid in a crystalline bcc matrix (Crystal + Liquid Pockets, ***XLP***) (Fig. 2D). The transition from an ***ISS*** to a ***XLP*** phase is a gradual process — the amount of dopants in the solid solutions decreases while the number of dopants part of a liquid-cluster increases over the range 0.01 < ***IF*** < 0.04 (Fig. 3). The liquid structure of the dopant pockets is corroborated by the calculated diffraction patterns, calculated separately for dopant-dopant and base-base structure, which show clear Debye-Scherrer rings associated with liquid order (Fig. 2F). These liquid pockets have internal volume fractions in the range of *ϕ*_*L*_ = 0.017 to *ϕ*_*L*_ = 0.04; below the volume fractions needed for vitrification, we therefor attribute the diffraction rings to a liquid and not to a glass phase. The presence of liquid clusters disrupts the bcc lattice of the matrix in such a way that the distinct Bragg reflections disappear from their diffraction pattern. The clusters appear to act as nuclei for grain boundaries; the diffraction pattern of the base crystal reflects its poly-crystalline nature. The grains still have an internal bcc symmetry as shown by the radial distribution function, *g*(*r*) and the distribution of the bond-orientational order parameter, ${\bar{q}}_{6}$ (See Supplemental Information, Figs S1,2 and Figs S3,4).

The distortion of the bcc matrix, upon increasing the amount of dopants and the emergence of liquid pockets, results in a superposition of scattering patterns with different spatial orientations (Fig. 2E and H) with excess Bragg peaks as a result. However, analysis of the bond-orientational order parameters ${\bar{q}}_{6}$ and ${\bar{q}}_{4}$ (See Supplemental Information, Figs S3–6) reveals that the local bcc symmetry is maintained, since their values are consistent with the body-centered cubic lattice and rule out other symmetries observed in these types of systems such as the face-centered cubic (fcc) or hexagonal close packed (hcp) lattices.

The evolution of an ***ISS*** towards stable pockets of dopant liquids is a unique feature of doped weak crystals. Recent work on hard sphere doped systems shows the co-crystallization of ***ISS*** phases with up to 14% of octahedral sites filled by dopants^29^; corresponding to ***IF*** ≈ 0.058 in our bcc systems. This is a bit higher than the point where all ***ISS*** dopants have disappeared and the system has fully converted to pockets of dopant liquid suspended in a bcc crystal, ***IF*** ≈ 0.04. In doped hard sphere fcc crystals at close-packing, the diffusion of dopants is effectively nullified. However, at slightly lower volume fraction there is dopant diffusion by hopping between interstitial sites, crossing a free energy barrier^10^. By contrast, the weak bcc lattice, *ϕ*_*base*_ = 0.1, allows for faster dopant diffusion when compared to a hard-sphere matrix.

**Figure 4:**
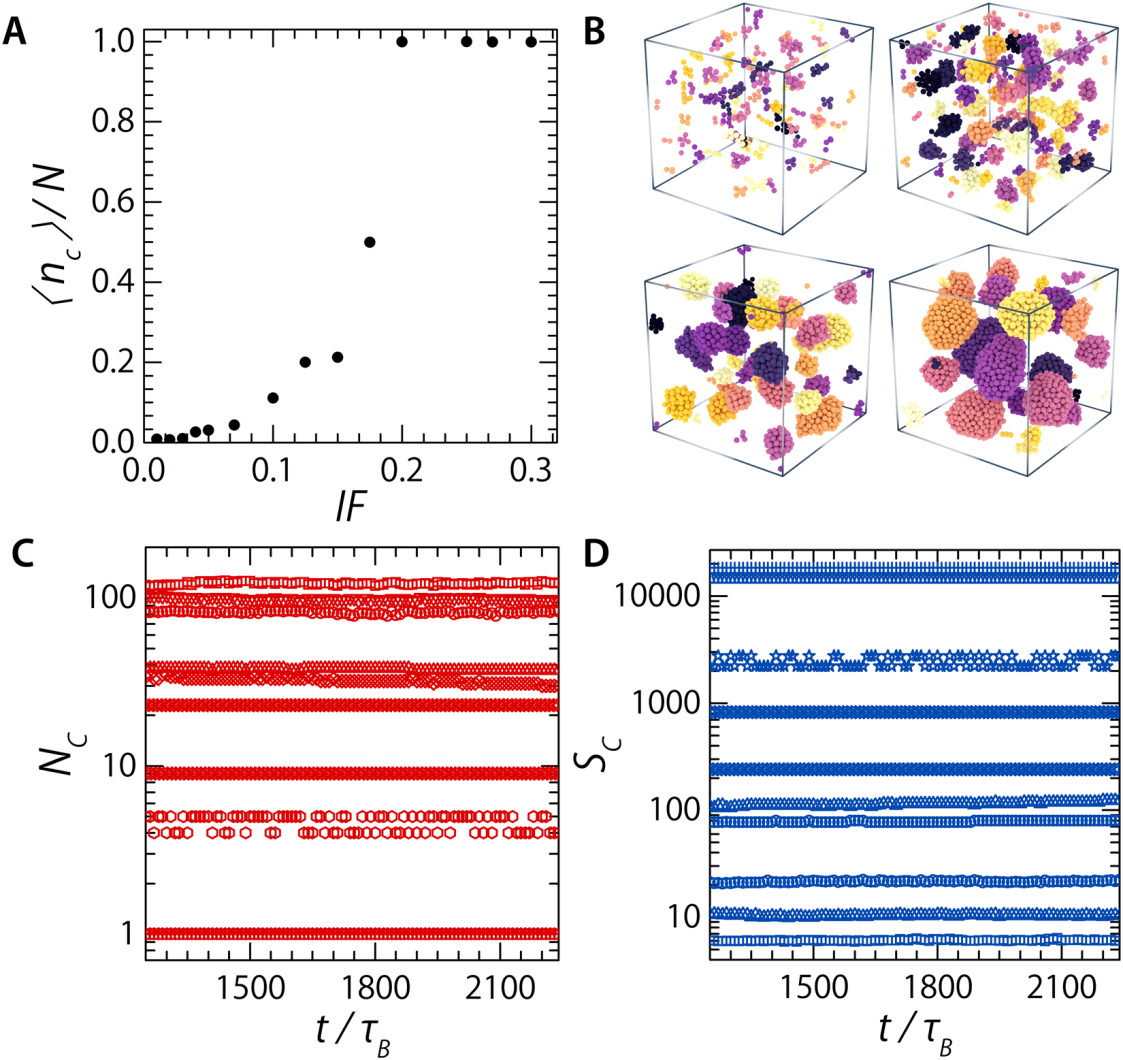
Number and stability of dopant clusters. (**A**) The number of dopant particles involved in a cluster divided by the total number op dopant particles, *n*_*c*_/*N*, as a function of the number of dopant particles; expressed as ***IF***. (**B**) Renderings of dopant clusters at the end of simulations for ***IF*** = 0.01, 0.03, 0.05, and 0.10 (**C**) Number of clusters present in a system for the last 1000 *τ*_*B*_ of the simulation. Different symbols represent different values for ***IF***. (**D**) Size of clusters, averaged over all clusters present in a system, for the last 1000 *τ*_*B*_ of the simulation. Different symbols represent different values for ***IF*** increasing from top to bottom for (**C**) and increasing for bottom to top for (**D**). Values of ***IF*** are those reported in the Methods section.

So far we have observed a transition of a interstitial solid solution into a phase where the dopant particles have phase separated into a number of clusters with a liquid internal structure — upon increasing ***IF*** from 0.01 to 0.04. As the amount of dopants increases further, ***IF*** ≥ 0.05, the size of the dopant clusters increases while their number decreases (Fig. 4) until the system separates into two distinct phases of a bcc matrix crystal of large particles and a liquid of dopants, a crystal-liquid coexistence ***XL***, ***IF*** > 0.10, the separated and kinetically stable pockets merge into a single phase (Fig. 2G, H, and I).

**Figure 5:**
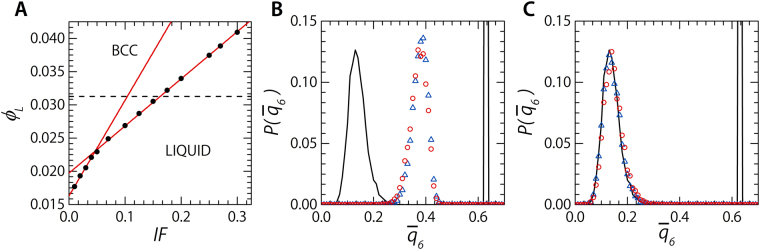
Volume fraction evolution of dopant phase. (**A**) The change in the local volume fraction, calculated via voronoi tessellations, of dopants belonging to the phase separated phase as a function of the number of filled tetrahedral interstitial sites. The red lines are linear fits to the two distinct behavioural regimes as explained in the text. The dashed line indicates the approximate melting point, *ϕ*_*m*_ of a dopant only system. (**B**) The distribution of the bond-orientational order parameter, ${\bar{q}}_{6}$, for a system at a volume fraction *ϕ*_*L*_ = 0.03220 (***IF*** = 0.175). Calculated for the sides of the system which at the start of the simulation were either a bcc crystal (red circles) or a liquid (blue triangles). The black lines indicate the original distribution of ${\bar{q}}_{6}$ at the start of the simulations. (**C**) The distribution of the bond-orientational order parameter, ${\bar{q}}_{6}$, for a system at a volume fraction *ϕ*_*L*_ = 0.03052 (***IF*** = 0.15). Calculated for the sides of the system which at the start of the simulation were either a bcc crystal (red circles) or a liquid (blue triangles). The black lines indicate the original distribution of ${\bar{q}}_{6}$ at the start of the simulations.

Over the entire ***IF*** range there is an increase in the internal volume fraction of the phase separated liquid dopant phase; ranging from *ϕ*_*L*_ = 0.01769 at ***IF*** = 0.01 to *ϕ*_*L*_ = 0.04092 at ***IF*** = 0.30 (Fig. 5A). This raises the question whether, in the phase separated dopant phases we are dealing with volume fractions above or below the melting point, *ϕ*_*m*_, of the equivalent system of only small dopant particles. To find the phase boundaries of a pure dopant system we simulate a system with as an initial condition a box filled half with a bcc crystal and half with randomised positions, at a fixed volume fraction. With this method, with both of the possible end configurations present at the start, we can easily determine which phase starts to dominate during the simulation and is thus the lowest energy state. We find the melting point for the pure dopant system at *ϕ*_*m*_ ≈ 0.03125 (Fig. 5A, dashed line). Below this point the system relaxes into a liquid while at *ϕ* > *ϕ*_*m*_ the system has a bcc crystal configuration as its equilibrium state. If we repeat the simulations with systems which have a fully liquid or crystal initial configuration we do not observe melting or freezing, suggesting that nucleation is rare and slow in these systems.

With the dopant freezing point at *ϕ*_*m*_ ≈ 0.031 the dopant phase in most of our simulations are in its liquid state. However, for those systems where *ϕ*_*L*_ > *ϕ*_*m*_, occurring for ***IF*** > 0.15, we would expect, based solely on the local volume fraction, to find a crystalline dopant phase. We observe different degrees of crystallinity in the dopant phase for ***IF*** = 0.20, 0.25, 0.27, & 0.30 based on their bond-orientational order parameter, ${\bar{q}}_{6}$ (See Supplemental Movies 1, 2, 3 and 4). This finding is corroborated by the probability distribution for the bond-orientational order parameter $P({\bar{q}}_{6})$; at these dopant fractions we observe a non zero probability of ${\bar{q}}_{6}\approx 0.4$, the expected value for a bcc symmetry (Fig. S4)^31^. The presence of local bcc order suggests that the dopant phase is definitely in a regime where it want to crystallise, but is hindered in doing so to the fullest extent; possibly by the irregularity if the dopant-base interface. The crossing of the dopant melting point introduces an new transition, from a crystalline matrix separated from a liquid dopant phase, ***XL***, into a meta-stable liquid dopant phase, ***XML***.

The rich phase behaviour of our system is summarised in Fig. 3. We plot the fraction of interstitial dopants and the fraction of separated dopants as a function of the total dopant concentration. We count both single dopants and clustered dopants with a cluster-averaged ${\bar{q}}_{6} > 0.4$ — indicating the cluster has bcc symmetry — as interstitial dopants. Two stages are observable. First the number of interstitial solid solution dopants (***ISS***; Fig. 3 example I) decreases as the total amount of dopants increases until we are left with a system where all dopants have phase separated from the base crystal in the form of liquid pockets (***XLP***; Fig. 3 example II). This first stage is followed by growth of the pockets until a single dopant phase has formed (***XL***), the doped system has at this point effectively separated into two phases. These distinct phases of separation and growth can also be observed in the increase of the local volume fraction of the phase separated dopants; there is a distinct kink at the point where the population of interstitial dopants ceases to exist (Fig. 5A points and solid lines). Finally the liquid dopant phase undergoes one more internal transition from a liquid into a meta-stable liquid (***XML***; Fig. 3 example III), with the occasional occurrence of a crystalline dopant phase (***XX***; Fig. 3 example IV).

The full phase separation of a binary system is predicted by thermodynamical models. However, the appearance of phase separated distinct clusters which are stable in time is unusual. We find that larger initial dopant concentrations lead to larger final clusters. For clusters in equilibrium we would expect that the cluster formation is reversible; lowering the number of dopants in a clustered configuration would result in the breakup of the larger clusters into smaller ones. To this end we start simulations from the final state of a simulation and remove a number of randomly selected dopants such that ***IF*** drops from 0.20, a single large cluster, to 0.10, multiple smaller cluster. For an equilibrium phase we would expect the large cluster to break-up into a multitude of smaller ones. This does not seem to be the case; instead the single large cluster compresses due to the expanding base crystal matrix (See Supplemental Information Fig. S7) — we are not dealing with an equilibrium phase but a kinetically trapped phase. To evaluate the stability of these kinetically trapped liquid clusters we measure the total number of clusters and the average size of those clusters over time. There are two possible coarsening modes for established clusters; either through Ostwald ripening, which would involve the transfer of particles from one cluster to another, or through coalescence — the merger of multiple cluster to form bigger ones. Coarsening through Ostwald ripening scales with the surface energy, *γ*_*d*,*b*_, between the dopant phase and base matrix. We calculate both the systems total potential energy, *U* and the surface area, *A* between dopant and matrix phases for the final 750 *τ*_*B*_ of our simulation; plotting the energy versus the area and fitting with a linear function, *U* ∝ *γ*_*d*,*b*_ *A* gives us an indication of the enthalpic surface energy in our system. We find *γ*_*d*,*b*_ = 4 *k*_B_ *T* · *σ* ^−2^ (see Supplemental Information Fig. S8), this low value on the order of *k*_B_ *T* makes it unlikely that Ostwald ripening is the driving force behind possible dopant droplet coarsening. The other option would be coarsening through merger events, this process relies on the ability of clusters to efficiently diffuse through the matrix in order to meet and merge. This seems highly unlikely, since all clusters appear to be kinetically trapped during our simulation (See Supplemental Information for cluster center-of-mass mean-squared displacements, Fig. S9). We therefore expect very little, if any, change in the average size of clusters once they are established. Indeed, during the last 1000 *τ*_*B*_ there is no discernible change in cluster numbers or average size (Fig. 4C and D).

**Figure 6:**
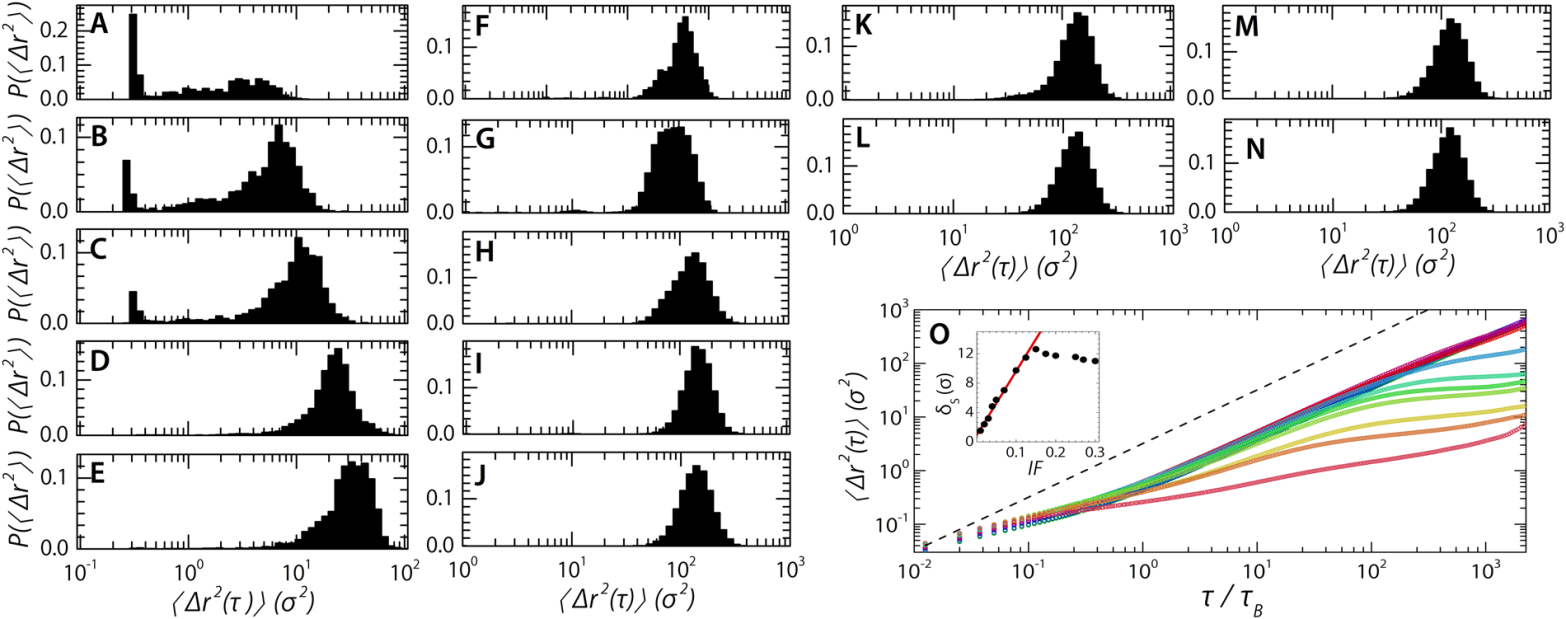
Mean-squared displacement of dopant particles. (**A**–**N**) Mean-squared displacement distributions, *P*(Δ*r* ^2^(*τ* = *τ*_*L*_)), with *τ*_*L*_ = 391 *τ*_*B*_ for ***IF*** = 0.01, 0.02, 0.03, 0.04, 0.05, 0.07, 0.10, 0.125, 0.15, 0.175, 0.20, 0.25, 0.27 & 0.30. Here angled brackets represent time averages. (**O**) Ensemble averaged dopant mean-squared displacement. Shown for, bottom to top, ***IF*** = 0.01, 0.02, 0.03, 0.04, 0.05, 0.07, 0.10, 0.125, 0.15, 0.175, 0.20, 0.25, 0.27 & 0.30. Here angled brackets represent time and ensemble averages. The dashed line is a line with a slope of one. (**inset**) Square root of the mean-squared displacement plateau height at *τ* = 391 *τ*_*B*_.

In doped crystals of hard spheres, where the volume fraction goes above fcc close packing, dopants diffuse trough the lattice at a much slower pace when compared to a weak bcc crystal^10,23^. The diffusion from one interstitial site to the next involves the crossing of an energy barrier set by surrounding base particles. In the case of a hard sphere fcc packing of the base crystal this energy barrier is on the order of several *k*_B_ *T* and has to be crossed twice because hopping goes trough an interstitial site of the opposite type, i.e. the transition from one octahedral site to the next goes through a neighbouring tetrahedral site. For weak bcc crystals the strongly increased fluctuations in the base crystal and the, in general, much lower volume fraction allows for far higher dopant diffusion rates when compared to an equivalent hard sphere system; in order to quantify this for our system we calculate the mean-squared displacement (MSD) of each dopant during the entire simulation after equilibration. We can classify the ensemble means of the dopant MSDs in roughly two categories; those systems where the dopants have separated into a number of finite-sized domains, the liquid pockets, and those where there is a single, continuous dopant phase present (Fig. 6O). Both domains have the expected vibrational displacements at short time-scales, *τ* < 0.3 *τ*_*B*_, where *τ*_*B*_ is the Brownian self-diffusion time of the larger base particles, and a diffusive regime at intermediate time-scales, *τ* ≈ 10 *τ*_*B*_. However, at longer times they differ. Whereas in the second category the diffusive regime persists for an extended period. Systems of the first category have a anomalous plateau at long times — the result of the finite dopant-domain size. The square-root of the height of this plateau represents the mean size of the dopant pockets, ${\delta }_{S}=\sqrt{{\rm{\Delta }}{r}^{2}(\tau =391\,{\tau }_{B})}$, which increases with increasing ***IF*** (Fig. 6O inset). In agreements with our visual observation of growing dopant cluster sizes with increasing ***IF*** (Fig. 4A). Of course, at infinitely long time-scales all MSDs should display purely diffusive behaviour with a slope of one due to the diffusion of entire dopant droplets. Running simulations long enough to capture this behaviour would take a prohibitively long time; a first indication of this upturn towards a slope of one can be seen in the curve representing ***IF*** = 0.01 (Fig. 6O bottom curve).

While the ensemble average MSD already gives us an indication of the behaviour of our doped crystals, the strength of our approach lies in the single-particle level information available. Therefore we also calculate the distribution of mean-squared displacements on a single particle level, *P*(Δ*r* ^2^(*τ* = *τ*_*L*_)) at long times, *τ*_*L*_ = 391 *τ*_*B*_. In these distributions there is reflected the same transition, from an interstitial solid solution phase into a phase consisting of separated liquid pockets, as we have previously seen in Figs 3 and 5. The indicator is the presence of a sharp peak in the MSD distribution in samples with low ***IF*** which represents dopant particles acting as interstitial dopants with an effective caging plateau around *τ* = 0.2 *σ* ^2^, where *σ* is the diameter of the larger base particles. (Fig. 6A–C). With the disappearance of this peak the distribution shift to higher values, coinciding with the up shifting of the confinement plateau described above (Fig. 6D–G). Using both the ensemble averaged and single mean-squared displacements we identify two transitions; i.e. the transition from an interstitial solid solution into a phase consisting of finite sized pockets of liquid dopant, followed by a second transition from finite-sized pockets towards a system spanning single dopant phase. The first transition occurs in the range 0.01 < ***IF*** < 0.04 with the second taking place between ***IF*** = 0.10 and ***IF*** = 0.125.

**Figure 7:**
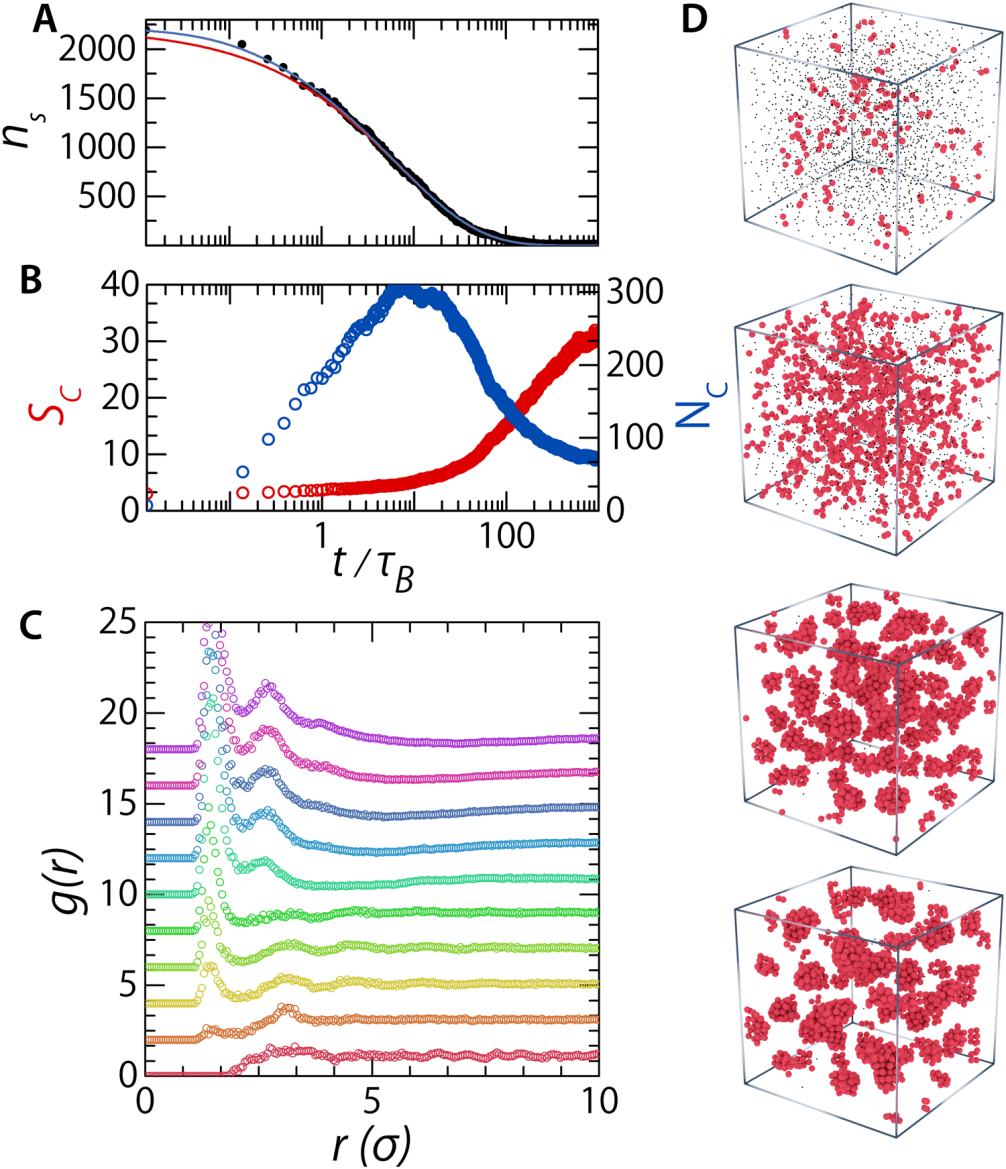
Time evolution of doped crystals. (**A**) Time evolution of the number of non clustered interstitial dopants, *n*_*s*_. The solid lines are fits with stretched exponential functions, both with *β* = 0.5 — Standard Kohlrausch decay function (red) and the modified Kohlrausch function (blue). (**B**) The change of the number of dopant clusters, *N*_*C*_ (blue curve), and average cluster size, *S*_*C*_ (red curve), with time. (**C**) Radial distribution functions, *g*(*r*), calculated for (from bottom to top) *t* = 0, 0.125, 1.25, 3.13, 12.5, 62.5, 125, 188, 375, and 994 *τ*_*B*_.

The high mobilities of dopants before clustering has taken place, especially when compared to binary hard sphere systems, in these weak bcc crystals is the determining factor which allows efficient lowering of the systems free energy by the formation of dopant clusters and pockets of liquid dopant. For metallic alloys phase separation proceeds by spinodal decomposition described by the Cahn-Hilliard theory^33–35^; where the origin of the new phase is non-nucleated demixing in the unstable regime. This is in contrast to nucleation and growth processes which feature small and well defined points of origin, nuclei, of the new phase which grow in time. To investigate the process of dopant phase separation we simulate an static doped base crystal at ***IF*** = 0.03. In this way the dopants can equilibrate throughout matrix. After equilibration, when all the dopants have distributed themselves over the available tetrahedral sites, we allow the base particles to move as well; we switch the system from a rigid to a weak crystal. At this point we also start recording positional data both of the base and dopant particles. Using this approach we can study the early kinetics of dopant droplet formation (Fig. 7D). The process of phase separation is visible in the radial distribution function, *g*(*r*), which we calculate for the dopants in the system. At *t* = 0*τ*_*B*_ we find no structural peaks and the *g*(*r*) resembles that of a gas indicating that our dopant form a solid solution. As time goes on we observe the appearance of structure peaks; including a shallow dip resulting from the finite size of the forming dopant droplets (Fig. 7C). Next we study the formed clusters in more detail. We find that at the first stages a lot of small clusters are formed which grow by the addition of single particles still present as a solid solution. This is illustrated by the appearance of a high number of clusters whose size is small (Fig. 7B *t*/*τ*_*B*_ < 5). Growth of the clusters on early timescales occurs by the addition of single dopants; the number of clusters stays constant during this time while their size increases slightly (Fig. 7B 5 < *t*/*τ*_*B*_ < 20). On longer time scales cluster-cluster combination events start to dominate — the number of clusters drops quickly while the average cluster size grows (Fig. 7B *t*/*τ*_*B*_ > 20). These processes continue until the cluster number and their average size becomes stable. The different growth modes lead to a changing growth rate over time. We can capture this behaviour by looking at the decrease of non-clustered dopants as a function of time (Fig. 7A). The rate at which this clustering process takes place changes over time; early times are dominated by growth due to addition of single dopants to existing clusters, while later times the clusters grow due merger events between clusters. Because of the changing rate constant we can not fit this curve with a simple exponential decay, instead we fit it with an stretched-exponential decay function (Kohlrausch function) with a time-dependant rate constant of the form, ${n}_{s}(t)={n}_{s,0}\exp (-k(t)\cdot t)$, with *n*_*s*_(*t*) the number of non-clustered dopants over time, *n*_*s*,0_ the number of single dopants at *t* = 0, and *k*(*t*) the rate constant of cluster growth which can be expressed as $k(t)=\frac{\beta }{{\tau }_{0}}{(\frac{t}{{\tau }_{0}})}^{\beta -1}$, with *β*, the stretching exponent, equal to $\frac{1}{2}$, and *τ*_0_ = 1.75 *τ*_*B*_ (Fig. 7A bottom red solid line). This functional form captures the observed slowing down of cluster growth due to the transition from the addition of free dopants to the growth by cluster merger events. However, it assumes that the point where the kinetics go from faster-than-exponential to sub-exponential is at *t* = *τ*_0_ and it has the problem that at *t* = 0 the rate constant is infinite. In our case it is not a certainty that the transition point lies at the exponential lifetime *τ*_0_; therefor we also try to fit an adjusted Kohlrausch function which has a rate constant that is always finite and decouples the transition point, *t*_0_, from the exponential lifetime, *τ*_0_. It has the from $k(t)=\frac{\beta }{{\tau }_{0}}{(\alpha +\frac{t}{{\tau }_{0}})}^{\beta -1}$ ^36^. where *α* = *t*_0_/*τ*_0_. This function describes our data well and captures the exponential decay rate at short times (Fig. 7A top blue solid line) for *τ*_0_ = 1.74 *τ*_*B*_, and *α* = 0.051. The small value of *α* confirms that, indeed, the first growth phase — single particle addition events — is very short compared to the later merger growth mechanism. The fact that a stretched exponential curve is required to describe cluster growth kinetics, implies that a distribution of characteristic reaction rates must exist. This is attributed to the cluster growth mechanism, which includes singlet addition to make a cluster grow with one dopant at a time, but the simultaneous cluster growth through cluster-cluster merger. If we presume the rate constant to be diffusion-limited, the reactivity of a distribution of species with different sizes, will inevitably result in a distribution in reaction rate constants, hence the stretched-exponential shape we observe is the result of a continuous sum of exponential decays^37^.

## Conclusion

Doped weak bcc crystals of charged colloids exhibited a complex phase diagram in which we identify several transitions. At low levels of doping the dopants behave as an interstitial solid solution; occupying the tetrahedral interstitial sites of the bcc matrix. In the case of a rigid base crystal this is effectively the same behaviour as has been observed for colloidal binary systems with hard sphere matrices, where dopants diffuse through the lattice; hopping between interstitial sites. However, in our system, where fluctuations of the matrix lattice are substantial, we observe anomalous behaviour. The weak matrix allows for free movement of dopants — no longer bound by well defined minima in the potential energy field. On top of that, the introduction of dopants with long ranged repulsive interactions into the crystalline matrix results in a finite lattice strain. even though particles are far apart. The ability of dopants to diffuse efficiently through our system allow to to efficiently lower its free energy by separating the dopants out from the bcc matrix; this lowers the finite lattice strain due to the presence of dopants. It is this clustering that drives the formation of dopant clusters and, at higher dopant concentrations, pockets of pure dopant liquid. The separation of dopants at supersaturated levels is also observed in doped metals such as carbon in *α*-Fe^6^. With the distinction that in the case of Fe the carbon precipitates in the form of Fe_3_C, whereas we form pure dopant phases. There also appears to be a preference for certain crystal planes to start forming carbon clusters before full precipitation^5^. We have not been able to observe any preference of this kind.

At even higher dopant concentration the finite-sized pockets merge and transition into one single dopant phase fully separated from the matrix; a crystal-liquid coexistence. Further increases in doping levels continuously increases the volume fraction of the dopant phase, which at some point crossing its freezing point. Even though volume fraction are higher that the freezing point the observation of crystalline structure in the dopant phase is rare. Our simulations are the in-silico model of the colloidal system with long-ranged Yukawa interactions made up of poly(methyl methacrylate) (PMMA) colloids^38,39^. This opens up the exciting possibility of studying the phase separation of binary colloidal systems in experiments using the highly charged Wigner crystal systems. By contrast, in hard sphere systems, kinetically-trapped configuration are more likely to appear; it remains unclear for hard spheres if demixing in polydisperse systems can occur on experimentally realistic time scales for the volume fractions required to induce freezing^40,41^.

Finally, we note that so far we have studied the structure and ensemble-averaged phase behaviour of these anomalous doped crystals. Since their dynamical properties, such as their vibrational density of states and mechanics, are also of relevance for the practical use of doped materials with a bcc symmetry, understanding how large thermal fluctuations and the resulting non-affinity at the particle scale effect their dynamics is most certainly of interest for future study. For example, it has been established that small amounts of defect in well-ordered solids can lead to the emergence of a Boson peak in the density of states, which is typically a feature associated with amorphous solids^32,42^. While it has recently been established that pure bcc crystals exhibit such anomalies^19^, it remains unclear what effect doping may have. For the future, it will be interesting to explore if these effects also emerge not due to structural defects but due to the addition of interstitial dopants in these bcc phases, as a means to manipulate their electronic and mechanical behaviour.

## Methods

### Simulations

We use Brownian Dynamics simulations, following the over-damped Langevin equation:

1 $$\frac{d{\overrightarrow{r}}_{i}}{dt}=\frac{1}{\gamma }[{\overrightarrow{F}}_{U,i}+{\overrightarrow{\xi }}_{i}]$$

where *γ* is the particle drag coefficient, ${\overrightarrow{F}}_{U,i}$ is the force acting on particle *i* resulting from all potentials and ${\overrightarrow{\xi }}_{i}$ is a random force with an average of zero, $\langle {\overrightarrow{\xi }}_{i}\rangle =0$, and a mean squared value, $\langle {|{\overrightarrow{\xi }}_{i}|}^{2}\rangle =6{k}_{B}T\gamma /\delta t$. The equation of motion is integrated using the HOOMD-Blue software package, which uses the integration described first by Snook^43^, drawing the random force term from a uniform distribution.

We perform the simulations on NVIDIA GeForce GTX 960 GPUs in single precision mode using the simulation package HOOMD-Blue version 2.0.3-7^44,45^.

Our simulation parameters are based on an experimental colloidal system of charged PMMA colloids interacting via long-ranged repulsive interactions^19,39^. We use particle diameters of *σ*_*b*_ = 1.8 *μm* and *σ*_*d*_ = 0.9 *μm* for the base crystal particles and dopants respectively. We normalise all distances with respect to *σ*_*b*_ such that *σ*_*b*_ = 1. The chosen particle sizes on the order of a micron make sure that our simulation system is accessible in experiments and the size ratio of *σ*_*d*_/*σ*_*b*_ = 0.5 is close to that of carbon dopants in iron.

The particles in our simulations all interact via a pair-wise repulsive Yukawa potential. It has been shown that this potential can capture the overall behaviour of experimental crystalline systems of these charged colloids^46^. We use a Yukawa potential of the form $U(r)/{k}_{B}T=\varepsilon \frac{\exp -\kappa r}{r}$ for *r* < *r*_*cut*_ and *U*(*r*)/*k*_*B*_ *T* = 0 for *r* ≥ *r*_*cut*_, where *r*_*cut*_ = 10 *σ*_*b*_ We use the same parameters as we used in our previous simulation work^23^ which have been mapped onto the experimental system’s phase behaviour: *κ* = 1.8 *σ*_*b*_, and *ε*_*b*,*b*_ = 713; assuming a constant surface charge density we arrive at *ε*_*d*,*d*_ = 227. We set *ε*_*d*,*b*_ = 470 for the cross interaction by taking the average of the homo-interactions.

We simulate with a time step of 2.5 · 10^−5^ *τ*_*B*_, with *τ*_*B*_ the self diffusion time of the base particles in an infinitely diluted system calculated as ${\tau }_{B}=\frac{{\sigma }^{2}}{{D}_{0}}$, with ${D}_{0}=\frac{{k}_{B}T}{6\pi \eta (\sigma /2)}$. We equilibrate the system for 10 · 10^6^ steps (2.5 · 10^2^ *τ*_*B*_) followed by a further 90 · 10^6^ steps (2.25 · 10^3^ *τ*_*B*_) during which we save dataframes every 500 steps.

Our primary simulations consist of 31,718 base particles, *N*_*b*_, with a variable amount of dopant particles, *N*_*d*_. We arrange the base particles on a perfect bcc lattice at a constant volume fraction *ϕ*_*base*_ = 0.1 and place the dopant particles in tetrahedral interstitial sites of said lattice before starting the simulations. We express the number of added dopant particles as the fraction of filled tetrahedral interstitial sites, ***IF***, in the base bcc lattice — we simulate ***IF*** = 0.01, 0.02, 0.03, 0.04, 0.05, 0.07, 0.10, 0.125, 0.15, 0.175, 0.20, 0.25, 0.27 & 0.30. These correspond to *N*_*d*_ = 738, 1477, 2216, 2954, 3693, 5540, 7387, 9234, 11080, 12927, 14774, 18468, 19945 & 22161. To find the melting point in a system consisting purely of dopant particles we generate a simulation box half-filled with a bcc lattice and half with randomised particle positions. We run these simulations for 10 · 10^6^ steps. We also simulate the formation of clusters in time by starting with a static bcc lattice with 20 percent of tetrahedral interstitial sites filled with dopants. We let the dopants relax for 10 · 10^6^ steps and follow this with 90 · 10^6^ steps during which we allow the base crystal to move as well; every 500 frames we save positional data.

### Data analysis

All data analysis is performed using Python in IPython (Jupyter) notebooks^47^; making use of the packages numpy, scipy, scikit-learn, and matplotlib^48–51^. For the identification of dopant clusters we use the DBSCAN algorithm as implemented in scikit-learn with the maximum allowable neighbourhood radius, *ε*, equal to the average neighbour distance — the first minimum in *g*(*r*) for each respective sample — and a minimal cluster size of 3 particles.

#### Calculation of bond order parameters

For the calculations of bond-orientational order parameters we call the BondOrderAnalysis program of Lechner^31^. This open-source software tool calculates three-dimensional orientational bond-order parameters based on spherical harmonics according to:

2 $${\bar{q}}_{l}(i)=\sqrt{\frac{4\pi }{2l+1}\sum _{m=-l}^{l}{|\frac{1}{{\tilde{N}}_{b}(i)}\sum _{j=0}^{{\tilde{N}}_{b}(i)}{q}_{lm}(j)|}^{2}}$$

for *l* = 6 and where ${\tilde{N}}_{b}(i)$ is the number of neighbours of particle *i* and particle *i* itself, and *q*_*lm*_(*k*) is defined as the Steinhardt bond-order parameter^52^:

3 $${q}_{lm}(j)=\frac{1}{{N}_{b}(j)}\sum _{k=1}^{{N}_{b}(j)}{Y}_{lm}({{\bf{r}}}_{jk}),$$

here *N*_*b*_(*i*) is the number of neighbours of particle *j*, and *Y*_*lm*_(**r**_*jk*_) is the Laplace’s spherical harmonic of the vector between particle *j* and particle *k*.

#### Calculation of *ϕ*_*L*_

For the calculation of the local volume fraction, *ϕ*_*L*_, of the dopant phases we employ Voronoi tessellation. For which we call out to the voro++ program^53^. The local volume fraction is defined as the ratio of the particle volume and the volume of its three-dimensional Voronoi cell, and subsequently ensemble-averaged:

4 $${\varphi }_{L}=\frac{1}{{N}_{p}}\sum _{i=1}^{{N}_{p}}[\frac{\frac{4}{3}\pi {r}_{i}^{3}}{{V}_{i}}]$$

where *N*_*p*_ is the number of particles in the population of interest, and *r*_*i*_ and *V*_*i*_ are the radius and volume of the enclosing Voronoi cell respectively of particle *i*.

### Data Availability

All data needed to evaluate the conclusions in the paper are present in the paper and/or the Supplementary. The datasets generated during and/or analysed during the current study are available from the corresponding author on reasonable request.

## Electronic supplementary material

Supplementary Information

Movie S1

Movie S2

Movie S3

Movie S4
