# Supplementary material for: Doping colloidal bcc crystals — interstitial solids and meta-stable clusters
Source: Sci Rep. 2017 Oct 3;7:12634. doi: 10.1038/s41598-017-12730-8 (PMC5626750; doi:10.1038/s41598-017-12730-8)
Supplement: Supplementary file 1 — Supplementary Information [file 41598_2017_12730_MOESM1_ESM.pdf]

Supplemental Information: *Doping colloidal bcc crystals — interstitial solids and meta-stable clusters*

Ruben Higler[1], & Joris Sprakel[1*]

[1]Physical Chemistry and Soft Matter, Wageningen University, Wageningen, 6708 WE, The Netherlands

*To whom correspondence should be addressed; E-mail: joris.sprakel@wur.nl.

1 *g(r)*

In order to analyse the static structure of our samples we calculate the radial distribution function, $g(r)$, for every ***IF*** and separately for the base particles (Fig. S1) and dopant particles (Fig. S2).

2 $\mathbf{P(\bar{q}_6)}$ and $\mathbf{P(\bar{q}_4)}$

For the two particle populations separately we calculate the bond orientational order parameter, $\bar{q}_6$ and $\bar{q}_4$, for every particle in the system. For the calculations we use the open-source BondOrderAnalysis (*54*) program and find nearest neighbours based on a cutoff distance r_{cut} equal to the first minima in $g(r)$ for each respective sample and particle type. We plot the probability distribution, $P(\bar{q}_6)$ and $P(\bar{q}_4)$, for the larger matrix particles (Fig. S3 and S5) and for the smaller dopant particles (Fig. S4 and S6).

The distributions of the base particles show bcc crystalline symmetries over the entire ***IF*** range; as indicated by the peak around $\bar{q}_4 = 0.4$ and $\bar{q}_4 = 0.05$. This peak broadens in the ***IF***

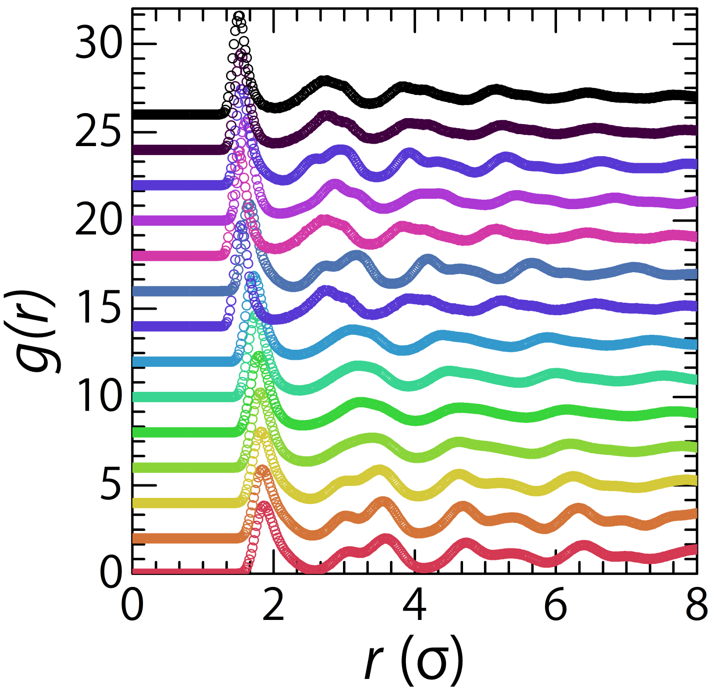

Fig. S1. Radial distribution function, $g(r)$, for base particles. Calculated for (bottom to top) ***IF*** $= 0.01$, 0.02, 0.03, 0.04, 0.05, 0.07, 0.10, 0.125, 0.15, 0.175, 0.20, 0.25, 0.27 $\&\, 0.30$.

regime ($0.01 <$ ***IF*** < 0.10) where there is a coexistence of dopant droplets suspended in a bcc matrix, and sharpens again when full phase separation takes place (***IF*** > 0.10).

The distributions of the dopant particles show broad distribution of $\bar{q}_6$ and $\bar{q}_4$ for ***IF*** < 0.04. This is because there are two populations of dopant particles; one which act as interstitial dopants (peak around $\bar{q}_6 >= 0.4$ and another where particles behave as a phase separated liquid ($\bar{q}_6 < 0.4$). For $0.04 <$ ***IF*** < 0.10 the distribution is dominated by dopant liquid, until traces of crystallinity reappear at those ***IF*** values where the system is fully phase separated and the dopant phase has a volume fraction above its freezing point (***IF*** > 0.10).

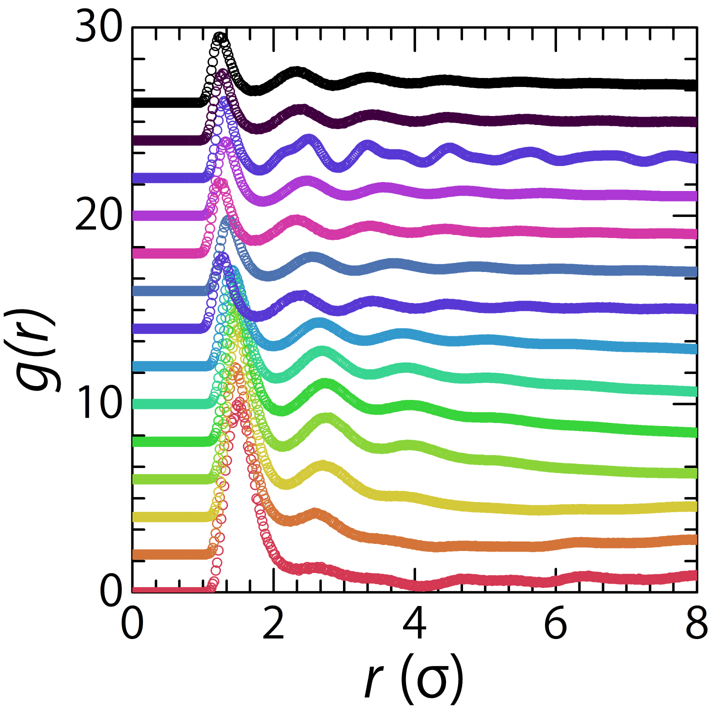

Fig. S2. Radial distribution function, $g(r)$, for dopant particles. Calculated for (bottom to top) ***IF*** $= 0.01$, 0.02, 0.03, 0.04, 0.05, 0.07, 0.10, 0.125, 0.15, 0.175, 0.20, 0.25, 0.27 & 0.30.

3 Reverse simulations

To investigate the thermodynamical reversibility of the phase separation we perform a reverse simulation. We use the final state of our simulation at ***IF*** $= 0.2$ as the initial configuration for a new simulation. Before starting the simulation we remove a number of random dopant particles such that the new ***IF*** $= 0.1$ (Fig. S7a). We than start the simulation and observe the behaviour of the dopant phase. Instead of the big dopant phase breaking up into smaller droplets, which we would expect for a reversible process as at ***IF*** $= 10$ we find separate dopant droplets, the phase simply undergoes isotropic compression by an expanding matrix phase until both phases find a new pressure equilibrium (Fig. S7b).

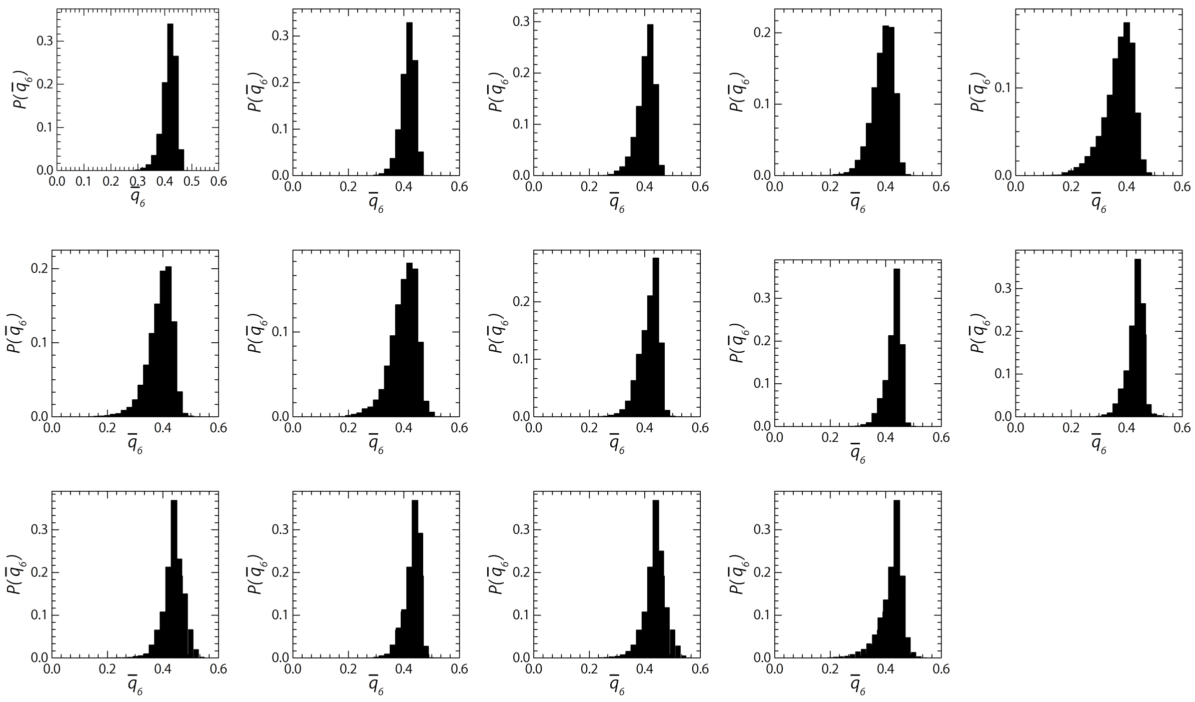

Fig. S3. Probability distribution of the bond-orientational order parameter, $\bar{q}_6$ for base particles. Calculated for samples with (left to right; top to bottom) ***IF*** $= 0.01$, 0.02, 0.03, 0.04, 0.05, 0.07, 0.10, 0.125, 0.15, 0.175, 0.20, 0.25, $0.27\,\&\,0.30$.

4 Interfacial enthalpy

In order to aid in the analysis of the stability of the clusters we calculate an interfacial enthalpy between the dopant and base phases, $\gamma_{b,d}$ term; similar to an interfacial energy, $\gamma_{b,d} \propto \frac{A_{b,d}}{U}$, where $A_{b,d}$ is the area between the dopant and base phases and U is the total potential energy of the system (Fig. S8).

5 Dopant droplet mean-squared displacement

Over the last 20 τ_B of our simulation (for ***IF*** $= 0.07$) we calculate the mean-squared displacement of entire dopant droplets by treating the centre-of-mass of each droplet as a particle and tracking its position over time. We observe rattling at short lag times which quickly turns into a

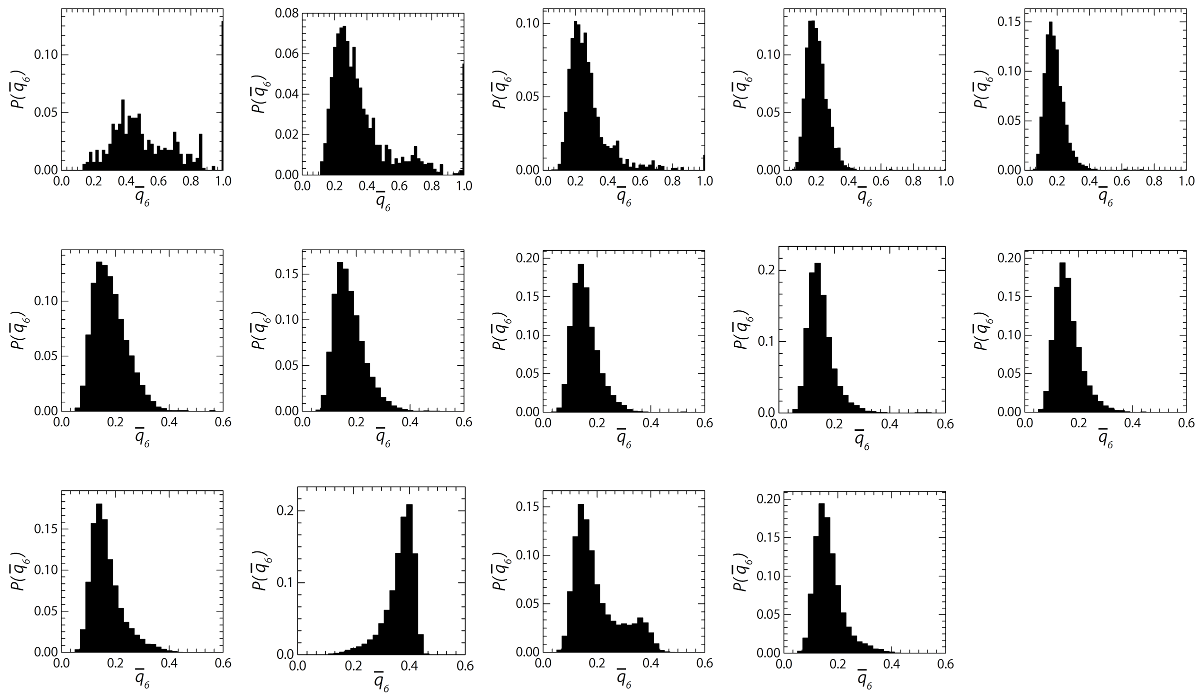

Fig. S4. Probability distribution of the bond-orientational order parameter, $\bar{q}_6$ for dopant particles. Calculated for samples with (left to right; top to bottom) ***IF*** $= 0.01$, 0.02, 0.03, 0.04, 0.05, 0.07, 0.10, 0.125, 0.15, 0.175, 0.20, 0.25, $0.27 \,\&\, 0.30$.

plateau at longer lag times. This indicates that dopant droplets are immobile and appear kinetically trapped (Fig. S9).

6 Supplemental Movies stills

These are still frames from Supplemental Movies 1-4 showing the localised amounts of crystallinity in the dopant phase. For ***IF*** $= 0.20,\ 0.25,\ 0.27,\ \text{and}\ 0.30.$

References

54. W. Lechner, C. Dellago, *The Journal of Chemical Physics* **129**, 114707 (2008).

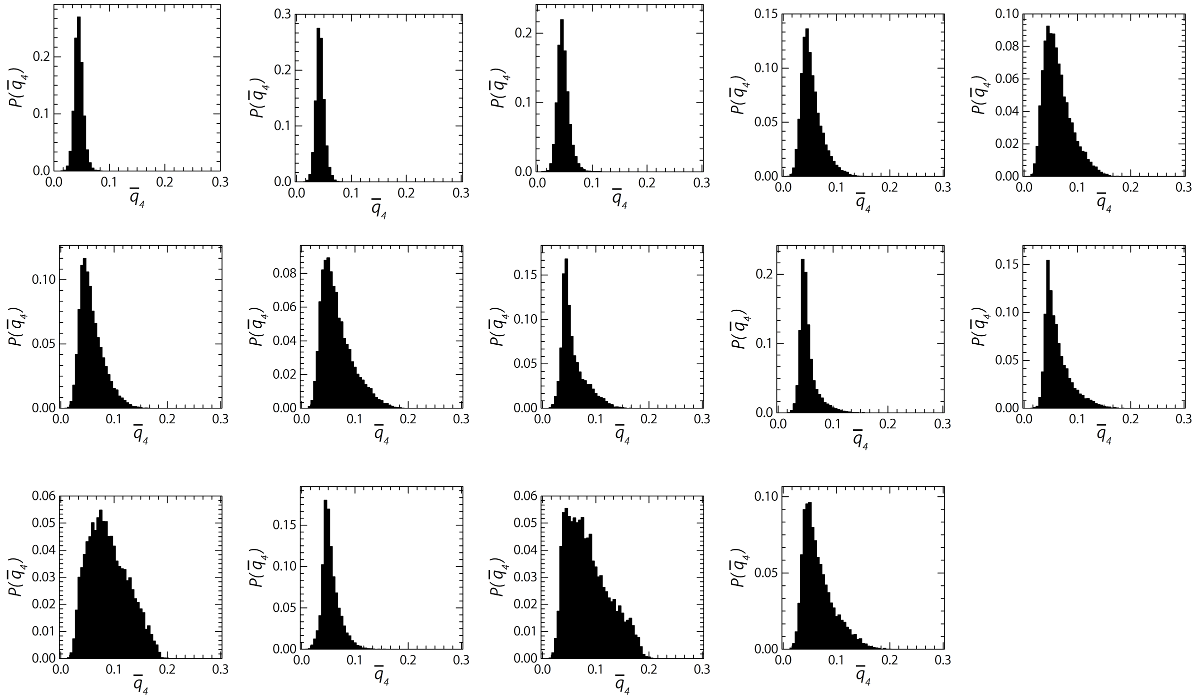

Fig. S5. Probability distribution of the bond-orientational order parameter, $\bar{q}_4$ for base particles. Calculated for samples with (left to right; top to bottom) ***IF*** $= 0.01,\ 0.02,\ 0.03,\ 0.04,\ 0.05,\ 0.07,\ 0.10,\ 0.125,\ 0.15,\ 0.175,\ 0.20,\ 0.25,\ 0.27\,\&\,0.30$.

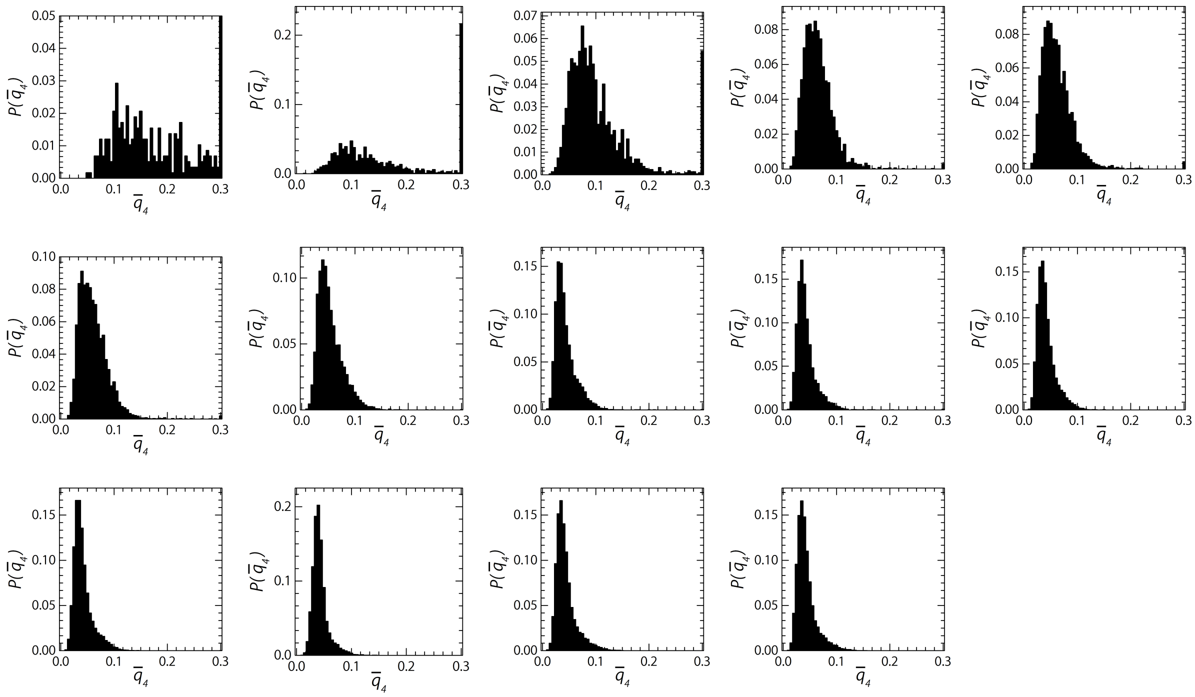

Fig. S6. Probability distribution of the bond-orientational order parameter, $\bar{q}_4$ for dopant particles. Calculated for samples with (left to right; top to bottom) ***IF*** $= 0.01$, 0.02, 0.03, 0.04, 0.05, 0.07, 0.10, 0.125, 0.15, 0.175, 0.20, 0.25, $0.27\,\&\,0.30$.

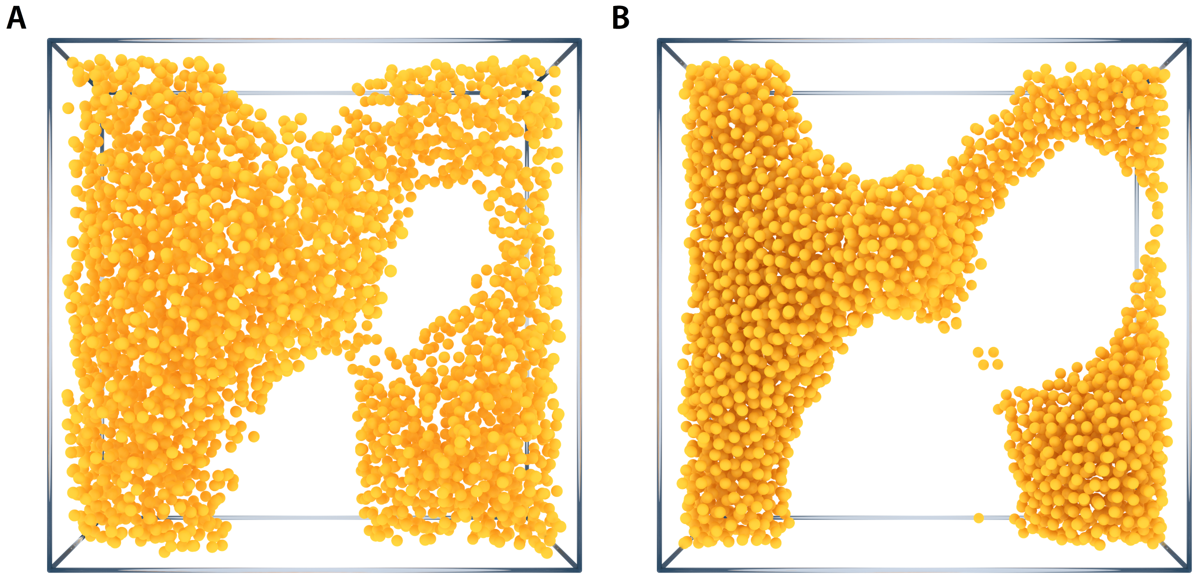

Fig. S7. Reverse simulations for *IF* $= 0.20$. (a) The initial configuration of the reverse simulation — the final state of the simulation at ***IF*** $= 0.20$ minus a number of random of dopant particles such that ***IF*** becomes 0.10. (b) Final state after the reverse simulation, clearly showing the isotropic compressed dopant phase.

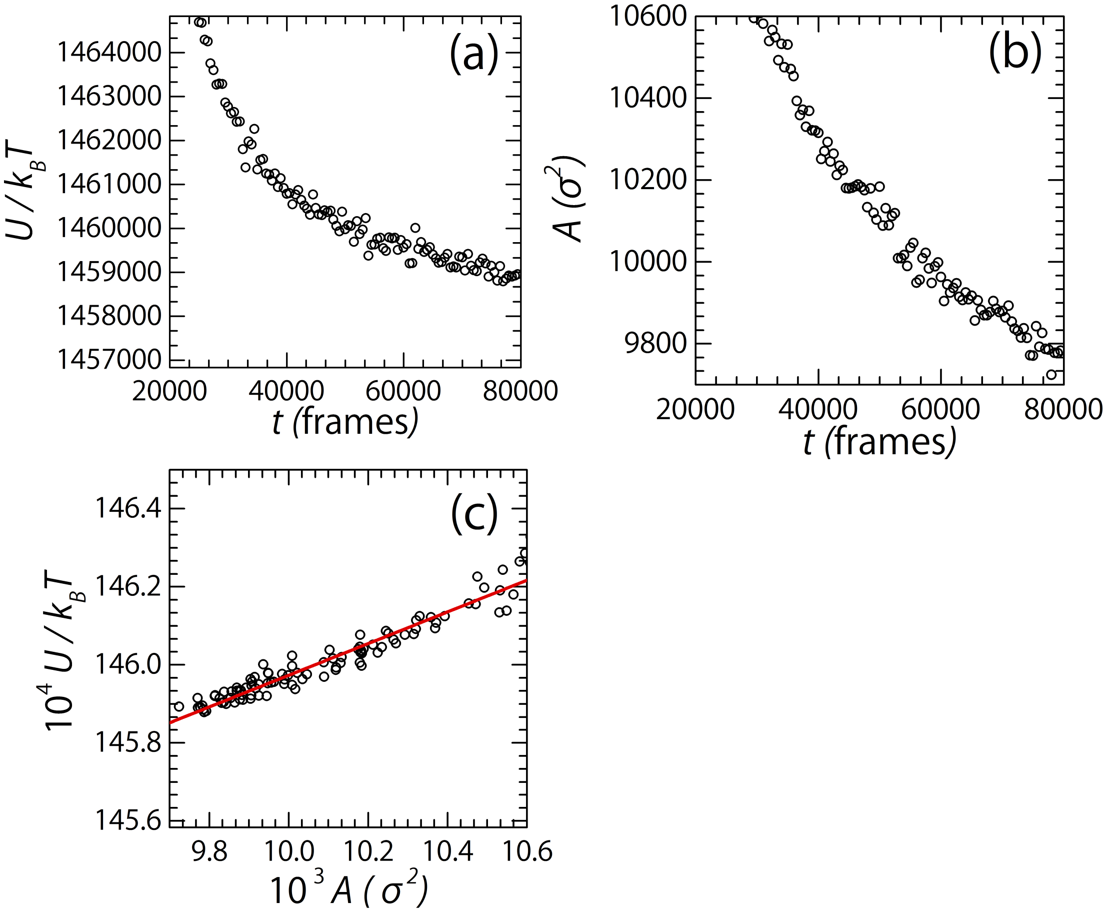

Fig. S8. Interfacial enthalpy between dopant and base particle phases. (a) Total potential energy of the system, U/k_BT. (b) Total interfacial area between dopant and base particles phases, in units σ^2, where σ is the diameter of a base particle. (c) Plot of total potential energy versus interfacial area. The solid line is a linear fit which gives us the enthalpic interfacial energy, $\gamma_{d,b} \propto \frac{A_{b,d}}{U}$, where $A_{b,d}$ is the area of the interface and U is the systems total potential energy.

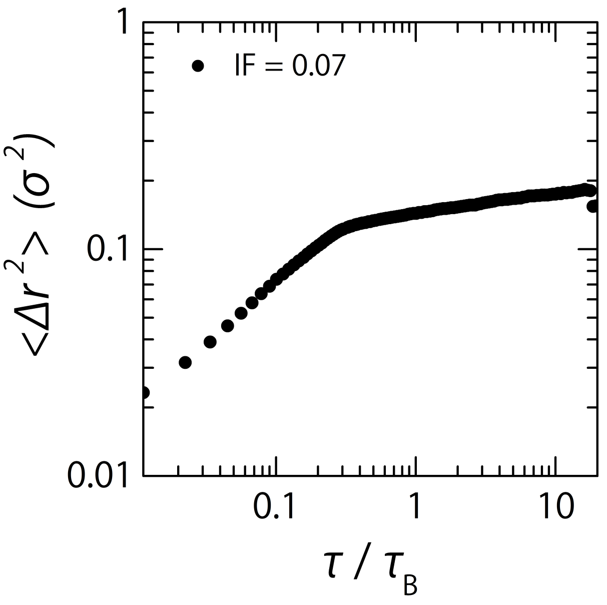

Fig. S9. Mean-squared displacements for dopant droplets. Calculated over the last 20 τ_B of our simulations for ***IF*** $= 0.07$.

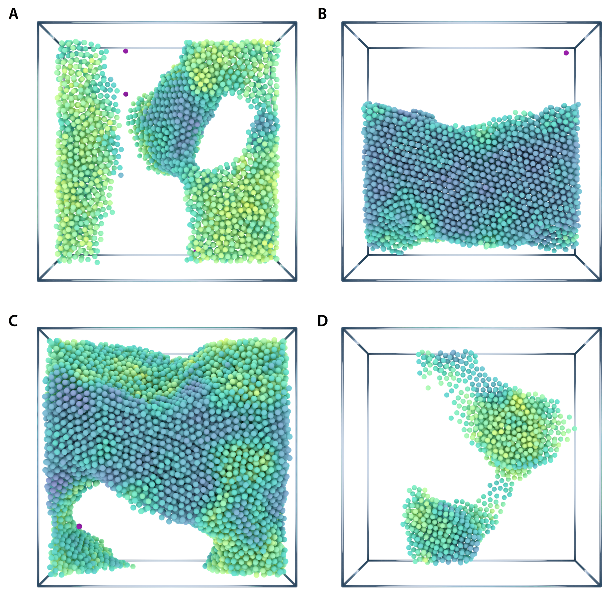

Fig. S10. Stills from supplemental movies 1-4. (a) Movie S1, ***IF*** $= 0.20$. (b) Movie S2, ***IF*** $= 0.25$. (c) Movie S3, ***IF*** $= 0.27$. (d) Movie S4, ***IF*** $= 0.30$. The dopant particles are coloured by their bond-orientational order parameter $\bar{q}_6$, where dark shades indicate $\bar{q}_6$ values around 0.4 (bcc symmetry) and lighter shades represent $\bar{q}_6$ values around 0.2 (isotropic liquid symmetry).
